# Design and Implementation of an Atmospheric Anion Monitoring System Based on Beidou Positioning

**DOI:** 10.3390/s21186174

**Published:** 2021-09-15

**Authors:** Jinhu Wang, Binze Xie, Jiahan Cai, Yuhao Wang, Jiang Chen, Muhammad Ilyas Abro

**Affiliations:** 1 Collaborative Innovation Center for Meteorological Disaster Forecasting, Early Warning and Evaluation, Key Open Laboratory of Aerosol and Cloud Precipitation, Nanjing University of Information Science and Technology, China Meteorological Administration, Nanjing 210044, China; 20181204022@nuist.edu.cn (B.X.); 20181204001@nuist.edu.cn (J.C.); 20201248028@nuist.edu.cn (Y.W.); 20201248002@nuist.edu.cn (J.C.); 2 National Experimental Demonstration Center for Atmospheric Science and Environmental Meteorology, Nanjing University of Information Science and Technology, Nanjing 210044, China; 3 Nanjing Xinda Institute of Safety and Emergency Management, Nanjing 210043, China; 4 Education and Literacy College, Department Government of Sindh, Hyderabad 71000, Pakistan; mohammadilyas.abro@yahoo.com

**Keywords:** Beidou positioning, STM32, atmospheric anions, 4G, capacitance suction method, unmanned driving

## Abstract

Atmospheric oxygen anions play an important role in medical health, clinical medicine, environmental health, and the ecological environment. Therefore, the concentration of atmospheric anions is an important index for measuring air quality. This paper proposes a monitoring system for atmospheric oxygen anions based on Beidou positioning and unmanned vehicles. This approach combines Beidou positioning technology, 4G pass-through, the unmanned capacitance suction method, electromagnetic field theory, and atmospheric detection technology. The proposed instrument can monitor the overall negative oxygen ion concentration, temperature, and humidity in a certain region over time and provide data visualization for the concentration of negative oxygen ions.

## 1. Introduction

In recent years, the problem of environmental pollution, especially air pollution, has become increasingly prominent. With improvements in social productivity and the economic level, the negative impact of air pollution on China has gradually been observed, especially after China entered the new economic normal in recent years. Air pollution has gradually become a key issue that has affected China’s comprehensive development [1]. Humans live in the atmospheric environment, and the physical and chemical properties of the atmosphere are closely related to human health. Human industrialization activities have greatly disrupted the ion balance in nature. Exhaust from automobiles, various soot types emitted from factories, pollution during the incineration of garbage, pollution related to the use of pesticides and organic compounds, the destruction of the ozone layer by air conditioners and refrigerators, and the electromagnetic waves generated by various household appliances have led to increases in positive ion levels. The negative ions in the air have many benefits to the environment and human health [2,3,4,5,6,7]. Improving the ion balance can increase comfort and restore the body to a healthy state; therefore, the problem of negative ions has attracted considerable attention [8]. Unmanned devices have played many roles in environmental detection [9,10,11,12]. The level of negative oxygen ions is a good indicator of air quality, living environment quality, and ecological quality [13,14,15].

Oikonomou et al. [16] used principal component analysis (PCA) to highlight the ability of sensing systems to discriminate among gaseous environments with different compositions/concentrations. Kolarz et al. [17] proposed a method of using air ion measurements as high-confidence indicators of change in the radon concentration. Poma et al. [18] described the development and validation of an autonomous system for the remote monitoring of the pH and temperature of seawater. Jean et al. [19] used a newly developed method to prepare a reference material for atmospheric dust materials similar to PM2.5. Silvia et al. [20] determined several inorganic anions in the atmosphere.

In China, studies of atmospheric negative ions peaked in popularity in the early 1980s and again in the early 1990s. The atmospheric anion concentration has been listed as an important parameter that can be used to measure air quality in environmental assessments [21]. Maciej et al. [22] designed an atmospheric anion measurement instrument that uses a disc device with positive and negative electric field phases that can collect positive and negative ions in the atmosphere and generate current to measure the positive and negative ion levels in the atmosphere. Sekia et al. [23] used a gallium liquid metal ion source to scan and measure the positive and negative ions in the atmosphere. Most of the domestic measurement methods for atmospheric negative ions use instruments designed by the Zhangzhou Southeast Electronics Research Institute in Fujian Province; specifically, a capacitive air ion collector is used to determine the charges carried by the ions. The current and the sample air flow generated by the charge are used to obtain the ion concentration. However, the abovementioned ion measurement instruments are all manually operated and cannot be automated for measurement and transmission [24]. The common atmospheric anion instruments include the BECKETr and Moody portable air ionizers produced by the DEV Company in the United States and the KEC-8002 atmospheric ion meter and the ITC-201A atmospheric ion meter III produced in Japan. The domestic negative ion measurement instruments mainly include the DLY series produced by Fujian Lianteng Electronics Co. Ltd. and the Forest Tourism Research Center of Central South University of Forestry and Technology. The capacitive air ion collector collects the charges carried by air ions, measures the charge formation current and sampling air flow, and then calculates the ion concentration [24]. Fang et al. used deep reinforcement learning [25,26] studies and implemented a prediction model by using radar image data. Sunny et al. [27] used an installed Internet of Things based multipurpose monitoring system for a specific nuclear storage situation measuring hydrogen concentration and temperature. Jin et al. [28] presented a distributed predictor that can overcome unrelated data and sensor noise. Hua et al. [29] proposed a novel error correction of atmospheric pressure data observed with a mini-AWS based on machine learning. Liu et al. [30] studied the relationship between the concentration of negative ions and meteorological factors in Pukou, Nanjing.

At present, the self-developed devices made in China have problems such as insufficient measurement accuracy and poor data stability. Foreign R&D devices have high measurement accuracy. However, these devices are bulky, expensive, and difficult to commercialize. Therefore, an easy-to-point, portable, high-precision atmospheric negative ion detection device that monitors the status of negative oxygen ions in the regional atmosphere is needed. The device designed in this article provides ion collection, charge amplification, A/D conversion, temperature detection, humidity detection, OLED display, data processing, 4G data transmission, host computer display Beidou positioning, and unmanned driving functionalities. The material cost is about USD 200, which can meet the needs of society.

## 2. Overall System Design

**Figure 1:**
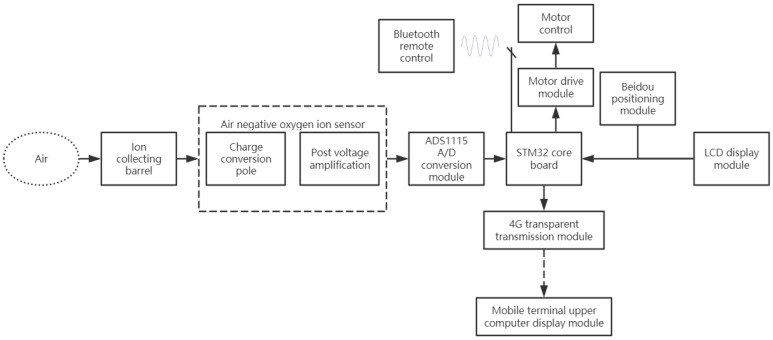
The overall design framework of the system.

The proposed system consists of a detection device and an unmanned device. The detection device is mainly composed of an STM32 F103ZET6, an ion collection cylinder, a high-precision AD conversion module, a charge amplifier module, and a 4G transparent transmission module. The unmanned driving device is mainly composed of the Beidou positioning module, motor driving module, motor module, and Bluetooth module. The Beidou positioning module is used to obtain longitude, latitude, time, altitude, and other information. The above modules are connected in a local area network. The computer on the computer side receives the data through the 4G transparent transmission module and displays the air parameter information on a map in real time. Moreover, the device can store the data. The overall design diagram of the system is as follows:

The hardware part in Figure 1 is mainly designed for the fixed-point detection of the concentrations of negative oxygen ions in the atmosphere. The Beidou positioning device combined with the unmanned driving module can display the monitoring information on the map of the mobile terminal. Additionally, the information obtained by the detection device can be uploaded to the computer through 4G for the data collector to perform subsequent data processing and real-time monitoring. The software system consists of a server and an intelligent software terminal for different software types. The system can perform data visualization based on multiple programming languages such as C# (Microsoft Inc., Redmond, WA, USA) and Java (Oracle Inc., Austin, TX, USA). The specific workflow is as follows:

(1) First, the Bluetooth controls the terminal and gives the command for the car to travel to a fixed monitoring point and stop at the point.
(2) The Beidou positioning module is used to obtain the longitude, latitude, altitude, and current time information for the current position, and the STM32 microcontroller transmits the data to the computer terminal via the 4G transparent transmission module.
(3) The negative oxygen ion collection bucket obtains negative oxygen ions in the air and transmits the data to the single-chip microcomputer for data processing by means of the charge voltage conversion module, voltage amplifier module, and AD conversion module. STM32 displays the data on the LCD screen and transmits it to the PC terminal computer for display by the 4G transparent transmission module.
(4) The temperature and humidity sensor works normally. The STM32 displays the temperature and humidity information on the LCD screen and transmits it to the PC terminal computer for display through the 4G transparent transmission module.
(5) Based on the Beidou positioning parameter information, the host computer terminal displays the position information of the monitoring system on the electronic map and displays the temperature, humidity, and negative oxygen ion concentration information for the monitored area.

The composition and functions of each software and hardware component are shown in Table 1. The accuracy of each sensor is shown in Table 2.

## 3. Hardware Module Design

### 3.1. Overall Scheme of the Hardware Module

The ion collection system uses a rectangular metal container as a shielding device, and a built-in cylindrical ion collection device is supplemented with a PWM speed-regulating fan to form an ion collection device. The ion collection device is connected to a charge amplifier, and the ADS1115 module transmits the collected voltage signal to the STM32 F103ZET6 microcontroller for data processing. During man–machine exchange, an LCD screen is used to display the negative oxygen ion concentration and other information in real time. The unmanned driving system under the ion collection system consists of a motor drive module, a motor module, a Beidou positioning module, and a Bluetooth module. The Bluetooth module controls the movement of the car, the Beidou module positions the car in real time, and the motor and the motor drive module drive the car forward. The data communication system uses a 4G transparent transmission module, which displays the data in real time on the host computer terminal and is marked on the map.

The ion collection system mainly includes a negative oxygen ion sensor module, a power module, a data conversion module, and a display module. The negative oxygen ion sensor system includes a metal shielding device, a cylindrical ion collection device, and a charge amplifier device. The cylindrical ion collection device is composed of a double-tube capacitor and a PWM speed-regulating fan. The power supply system includes a single power supply and a dual power supply. The modules include a boost module and a step-down module. The data conversion device is a 16-bit high-speed A/D conversion module, and the display device uses an LCD screen.

### 3.2. Hardware Module Terminal

#### 3.2.1. Detection Device

The detection device is composed of a cylindrical sensor, a charge amplifier, a DHT11 temperature and humidity sensor, a single power supply to dual power supply module, and an A/D conversion module. The cylindrical sensor absorbs air and collects negative oxygen ions in the air. When the charge amount is converted into a voltage amount by a charge amplifier, the AD conversion module converts the analog voltage amount into a digital amount and transmits this information to the microcontroller for data processing. Moreover, the DHT11 temperature and humidity sensor will collect information and then transmit it to the microcontroller for data processing.

**Figure 2:**
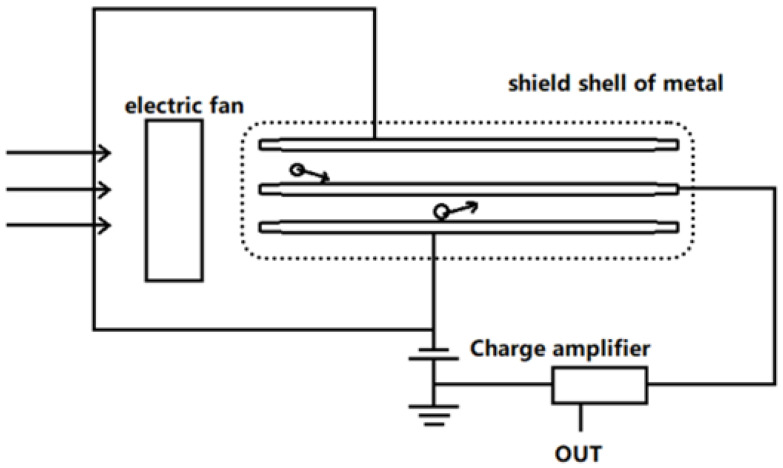
Structure of the cylindrical ion collection device (shield shell dimensions: 345 mm × 72 mm × 45 mm).

The cylindrical sensor device uses a capacitive suction method. The outer cylinder is a hollow metal barrel that is connected to a negative bias voltage. The inner cylinder is a solid cylindrical rod. When we start the PWM speed-regulating fan, the air is drawn into the double-barrel capacitor. Under the action of the bias voltage, the negative oxygen ions are deflected to the solid cylindrical rod, and then the solid cylindrical rod is connected to the charge amplifier by means of a wire and is grounded, as shown in Figure 2.

**Figure 3:**
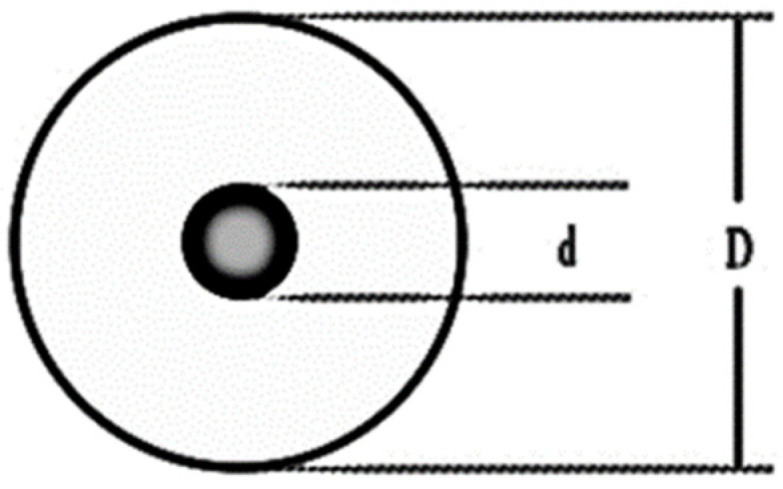
Side view of the structure of the cylindrical ion collection device (d = 5 mm, D = 20 mm).

A quantitative polarization voltage is loaded on the polarization plate of the ion sensor (collection tube), and then the measured air is transported through the sensor at a set speed. Specific small particle size negative ions in the air deflect under the electric field and are captured by the acquisition plate. After the processing of the negative charge amount of the collected negative ions, the charge concentration value of the negative ions can be calculated. By measuring the ion mobility K in cm^2^/(V·s), the negative ion concentration in the air can be calculated. Critical ion mobility K = 0.4 is used to measure small particle size negative ion concentration. Figure 3 is a schematic diagram of the ion collection device.

As the charge amplifier of the core component, the DCA10X charge amplifier module is a low-cost, high-precision DC charge amplifier module that can convert charge signals into voltage signals and outputs with good temperature stability, limited noise, and low frequency response error [31]. Its power consumption is 0.3 W. The amplifier uses a specially designed electrical zero circuit that is characterized by high accuracy and reliability and flexibly solves the problem of drift during low-frequency testing. The module is sealed and shielded with environmentally friendly resin, which has the advantages of moisture resistance, shock resistance, and anti-interference. The low static drift is suitable for static or dynamic charge measurement applications of >1 nC.

The circuit diagram of the charge amplifier is shown below.

**Figure 4:**
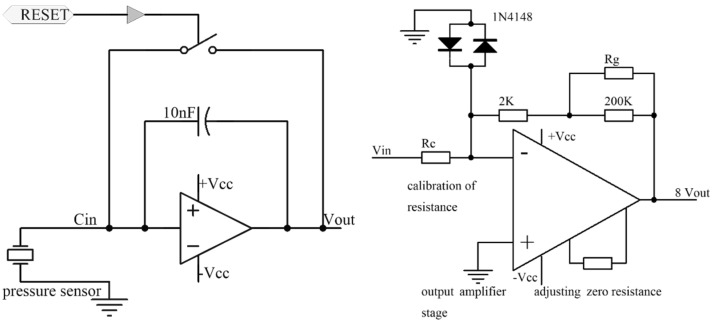
Charge amplifier circuit.

**Figure 5:**
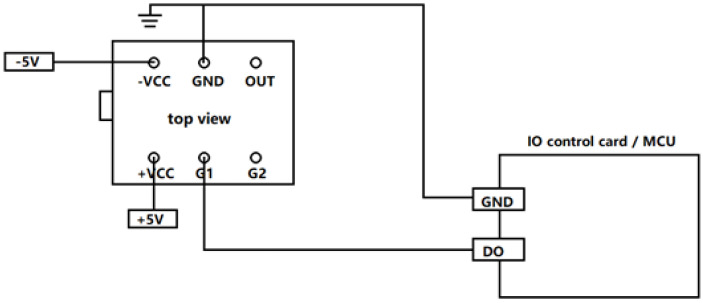
Connection diagram.

The charge amplifier shown in Figure 4 is composed of two parts: one is a charge conversion stage, and the other is a voltage amplification stage. The input range is ±220 to ±22,000 pC, and the output range is ±5 to ±10 V. R_g_ is an adjustable resistor that can control the voltage amplification factor. RESET is a control port. A pulse is sent from the MCU to control the charge conversion stage and maintain a value of zero. Then, preparation begins for the next charge collection. The charge amplifier and MCU are connected, as shown in Figure 5.

The output voltage of the charge sensor is set to *V*, and the unit is volts; the number of detected charges is N, the unit is one; the charge conversion rate is *K*, and the unit is mV⁄pC; *S* is the pumping speed, and the unit is L⁄s; and the density of negative oxygen ions in the atmosphere can be calculated as follows [32]:

(1) $$\rho_{-}=\frac{6.25\times 10^{8}\times V^{2}}{K\times S}\;$$

**Figure 6:**
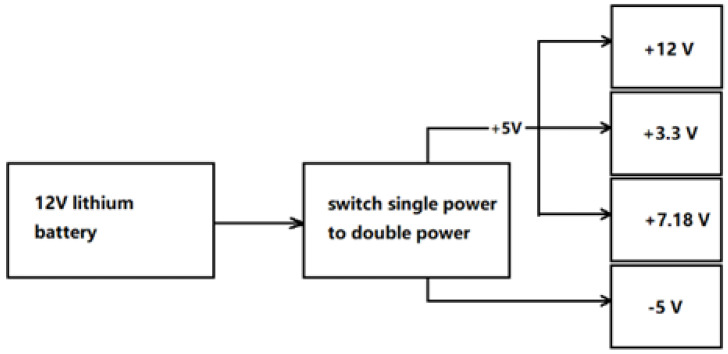
Power module.

A 12 V lithium battery is used for the power supply, and single-power to dual-power modules, step-up modules, and step-down modules are used to generate DC voltages of ±5, +12, and +3.3 V to power the modules of the device. The specific circuit is shown in Figure 6.

**Figure 7:**
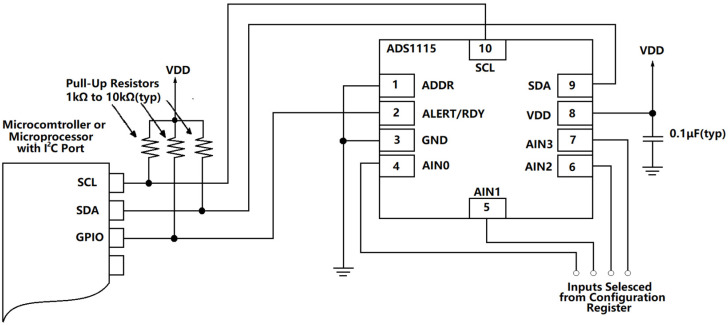
ADS1115 A/D conversion module.

In the circuit shown in Figure 6, the data transmission device uses a 16-bit high-precision ADS1115 A/D conversion chip, which can recognize millivolt- or even microvolt-level voltages [33]. Its continuous working current is extremely low, only 150 μA. This chip is controlled with the MCU, and the corresponding circuit diagram is as follows:

In the circuit shown in Figure 7, the display device uses ILI9341. Parallel communication data transmission is faster and more accurate than traditional transmission, thus facilitating subsequent development and improving functionality.

**Figure 8:**
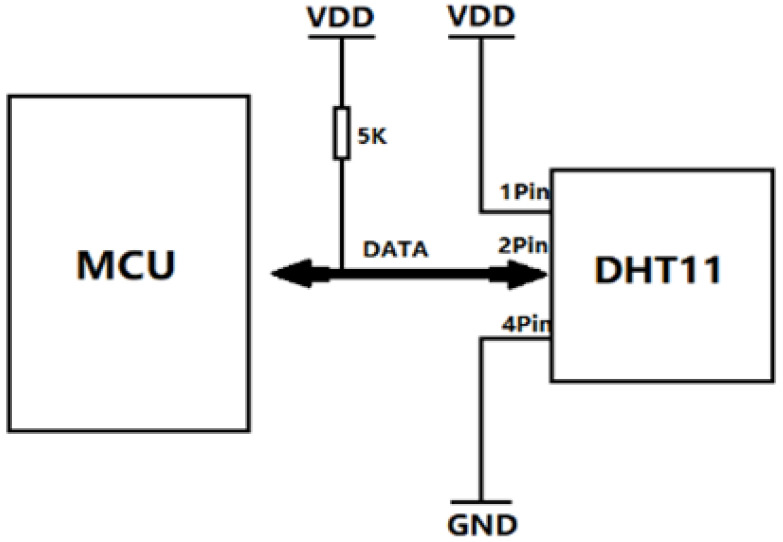
DHT11 temperature and humidity sensor.

**Figure 9:**
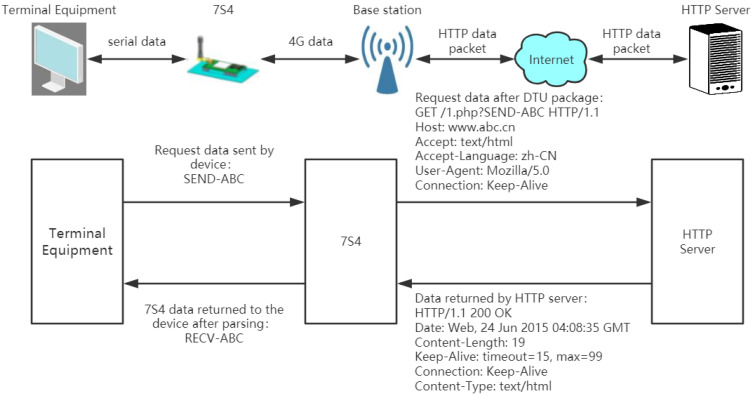
Detection information transmission process.

The DHT11 temperature and humidity sensor module is shown in Figure 8. The air-sensing device uses a DHT11 temperature and humidity sensor. The DHT11 digital temperature and humidity sensor is a temperature and humidity composite sensor with a calibrated digital signal output. Special digital module acquisition technology and temperature and humidity sensing technology are applied to ensure that the product has extremely high reliability and excellent long-term stability. The sensor includes a resistive humidity-sensing element and an NTC temperature-measuring element and is connected to a high-performance 8-bit microcontroller. Therefore, this product has the advantages of excellent quality, an ultrafast response, strong anti-interference ability, and low-cost performance. Each of the DHT11 sensors is calibrated in an extremely accurate humidity calibration chamber. The calibration coefficients are stored in the OTP memory in the form of a program, and these calibration coefficients are called by the sensor during the processing of the detection signal [34]. A single-wire serial interface makes system integration easy and fast. The ultrasmall size and extremely low power consumption make this device the best choice for harsh applications. The product is a four-pin single-row package for easy connection, and the circuit diagram is shown in Figure 9.

#### 3.2.2. Unmanned Terminal

The unmanned system is mainly composed of a motor drive module, motor module, Beidou positioning module, and Bluetooth module. The Bluetooth module controls the movement of the car, the Beidou behavior module locates the position of the car in real time, and the motor and motor drive modules move the car forward.

(1) The Bluetooth module utilizes the master-in-one Bluetooth serial port module and the Bluetooth control Arduino application to connect to the car’s HC-05 module. In short, when a Bluetooth device is successfully paired and connected with another Bluetooth device, we can ignore the internal communication protocol of the Bluetooth and directly use the corresponding serial port. When a connection is made, the two devices share a channel or serial port. One device sends data to the channel, and the other device receives data from the channel. The unmanned car, as the carrying platform of the negative ion detection device, uses the minimum function Bluetooth remote control car based on Arduino. Hardware includes tire, body, TT dual-shaft motor, L298N motor drive panel, Arduino master panel, and serial Bluetooth module. Bluetooth control can use BlueSPP software to download the app on the phone to connect to the Bluetooth device and send messages through a serial communication protocol.
(2) Motor and motor drive modules. The motor drive module adopts the L298N module, which includes four single-pole double-throw switches. The combination of various-level signals emitted by the microcontroller (the Arduino microcontroller is commonly used in DIY devices) is used to control the operation of the four switches. Thus, the motor is controlled, and the track of the car is controlled.
(3) Beidou positioning module. The Atkatk-s1216f8 Beidou module is a high-performance GPS Beidou dual-mode positioning module. The module includes the S1216F8S1216F module with a small size and excellent performance. The module can set various parameters through a serial port and can be saved in internal FLASH memory, which is easy to use. The module has an IPXIPX interface and can connect various active antennas. A connection to a GPS Beidou dual-mode active antenna is recommended. The module is compatible with signals at the 3.3 V/5 V level and is easy to connect in a variety of MCU systems. The module has a rechargeable backup pool that can keep historical data when the device power is off.

#### 3.2.3. Data Communication Terminal

As shown in Figure 9, USR-LTE-7S4 is a compact and feature-rich M2M product suitable for mobile Telecom 4G and China Unicom 3G and 2G network standards. With “transparent transmission” as the core concept, this product is easy to use, and users can easily and quickly integrate it into their own systems. The software used by this module provides numerous functions and covers most common application scenarios. Only via simple settings can two-way data transmission from a serial port to a network be realized. In addition, this module supports custom registration packages, heartbeat package functionality, four-socket connections, and transparent cloud access. Moreover, the module is characterized by high speed and low latency and supports other FTP upgrade protocols and FTP self-upgraded protocols. USR-LTE-7S4 is pin-compatible with the 2G products 7S2 and 7S3. Users can directly replace the product to achieve a substantial increase in the communication speed. A watchdog is included with the hardware to ensure the stability of the product for a long time. The computer uses a virtual serial port to receive 4G transparent transmission module data.

## 4. Software Design

### 4.1. Overall Plan of the Software Module

The system software consists of process control software, mobile terminal data acquisition software, and embedded software. The process control software provides certain main functions, such as controlling unmanned driving, and adopts Bluetooth point-to-point communication technology. The mobile terminal data acquisition software mainly provides functions such as data acquisition, data transmission, and visual processing to achieve the collection of negative oxygen ion concentrations in the atmosphere and support human–computer interactions. With the support of the Beidou positioning system, the hardware system obtains location information, atmospheric negative oxygen ion concentration information, and temperature and humidity information and transmits the data to mobile terminals to obtain area monitoring distribution maps via 4G transmission. The embedded software is mainly housed in the single-chip microcomputer chip to realize full hardware functionality.

### 4.2. Embedded Software Programs

**Figure 10:**
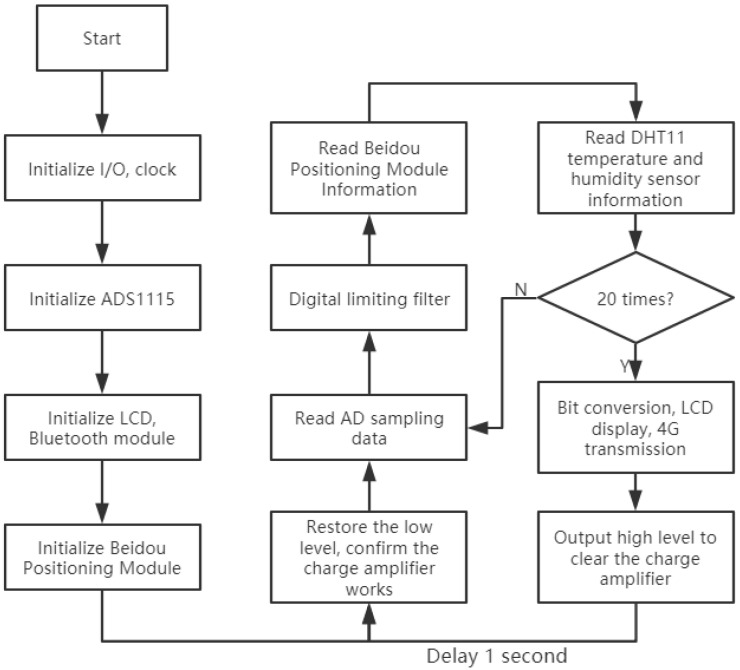
Overall program flowchart.

As shown in Figure 10, the system first initializes the pin clocks of each module and then performs register configurations on the A/D conversion module ADS1115, Bluetooth module, and LCD module. After the data from the negative oxygen ion sensor are sampled by AD, the data are clipped and filtered. Then, the filtering method is determined by assessing the difference between the two measured values. If the difference between the current value and the previous value is less than or equal to the limit value, the current value is valid. If the difference between the current value and the last value is greater than the limit value, then the current value is invalid. In such a case, the current value is discarded, and the previous value is used instead of the current value. The advantage of this approach is that it can effectively overcome the impulse interference caused by accidental factors [35].

During data processing, the program first initializes each module and then enters the loop structure. In the loop structure, the MCU first outputs a low value to confirm the operation of the charge amplifier and then reads the value of ADS1115 20 times for filtering calculations. The temperature and humidity sensor displays each value that is read, and the ADS1115 displays the converted results every 20 collection steps. Then, the MCU outputs a high value, and 1 s is needed to clear the charge amplifier and start the next cycle.

### 4.3. Process Control

**Figure 11:**
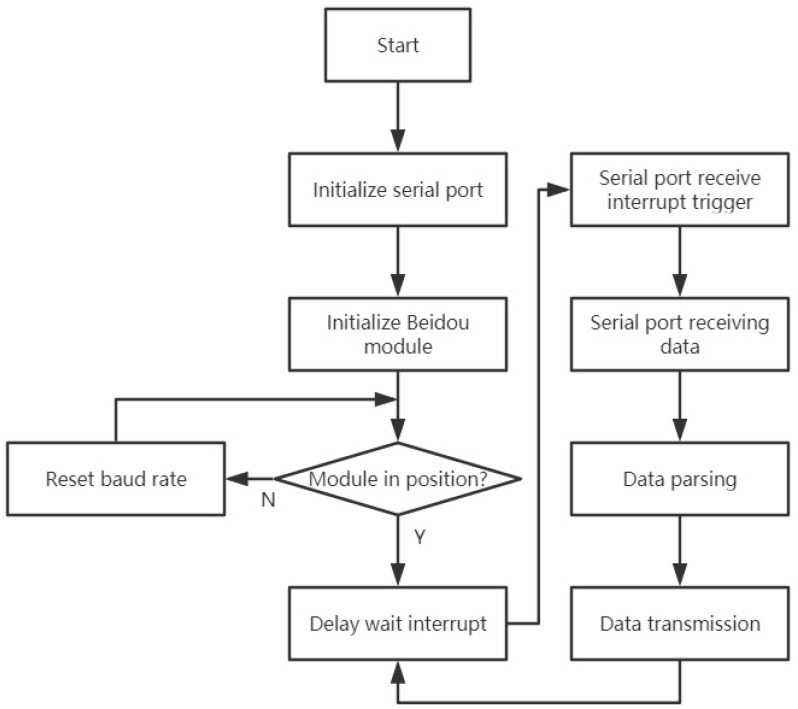
Beidou module program flowchart.

As shown in Figure 11, the Beidou module is used to obtain the longitude, latitude, and altitude information for the device. The Beidou data and the measured meteorological data are used to analyze atmospheric anion concentration information at different locations in the studied area. The Beidou module communicates with the controller through the serial port protocol. The Beidou module configuration and data processing steps can be completed through the serial port. The process is shown in Figure 11.

### 4.4. Mobile Terminal Data Acquisition Software

**Figure 12:**
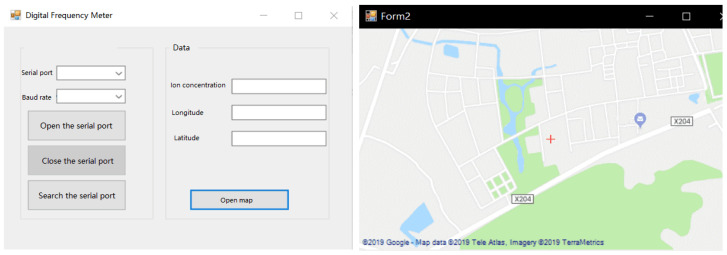
Upper computer display.

The upper computer display is shown in Figure 12. The upper computer display module consists of data and map displays. The map display uses C# to call GMAP, utilizing GMap. NET control, which loads maps of the company, such as Google or ArcGIS (Esri Inc., Redlands, CA, USA), based on the HTTP protocol, obtains the corresponding slice under map by parsing the URL and the incoming parameters of the company’s map service. Traditional costly map engine-dependent solutions have been solved for easy migration [36]. The current solutions, which involve Mapxtreme, ArcEngine, SuperMap, etc., are expensive. The deployment is complex and not suitable for the development of conventional desktop GPS monitoring systems. The data display is developed using the C# language, and the interface is simple and intuitive.

## 5. Equipment Test

**Figure 13:**
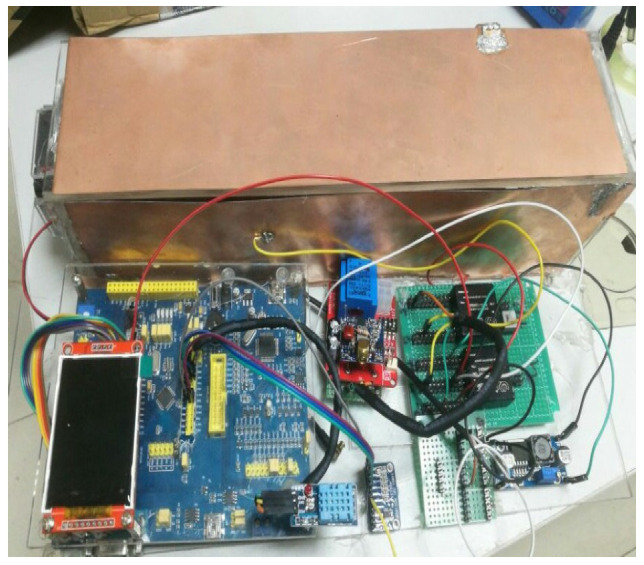
Appearance of the equipment.

The appearance of the equipment is shown in Figure 13. The picture shows an experiment conducted in Pukou District, Nanjing. The experimental results are compared with the results of Liu et al. [30], and the data trends are basically consistent.

**Figure 14:**
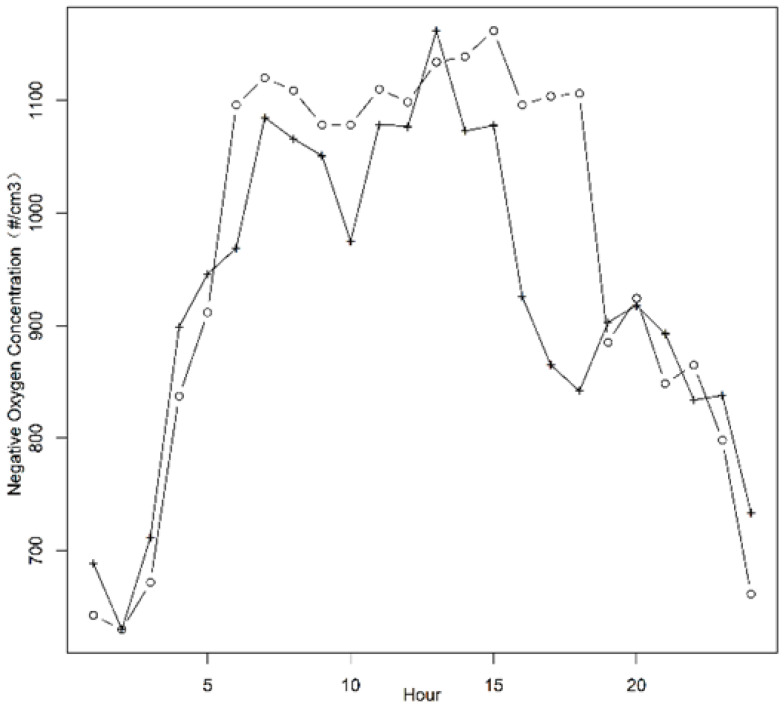
Negative oxygen ion concentration test (‘○’ is measured by this equipment and ‘+’ is measured by COM-3500A).

The negative oxygen ion concentration of the Nanjing University of Information Science and Technology was measured with this equipment and compared with the measurement result of COM-3500A. The result is shown in Figure 14.

The negative oxygen ion concentration began to rise from 2 o’clock to 7 o’clock and reached its peak value at about 1100 cm^−3^ from 14 to 15 o’clock. It began to decrease after 13:00 and reached its lowest value at 2 o’clock, about 650–680 cm^−3^.

Through comparison, it can be found that the observation results of the two sets of different equipment are very consistent, and the changing trends of the two sets of data are consistent and have a high correlation, but the test value of this equipment at low concentrations is relatively high.

## 6. Conclusions

This project applies Beidou positioning to the meteorological field and combines Beidou application technology with atmospheric detection and unmanned equipment. This approach takes advantage of Beidou positioning to achieve unmanned and intelligent technology, as well as contributing to the Internet of Things. Compared with large-scale negative oxygen ion monitoring equipment, this device is small, highly flexible, and cheap, and it consumes little power. It has been verified that the stability of the equipment and the accuracy of the data are within a reasonable error range, which can meet the needs of society.

## Figures and Tables

**Table 1:** Composition and functions of each software and hardware component.

Device	Sensors	Main Function
Detection device	Cylindrical sensor Charge amplifier Temperature and humidity sensor Digital to analog conversion module Power module	The sensor collects the negative oxygen ions in the air, converts the analog quantity into digital quantity after passing through the charge amplifier and A/D conversion module, and processes the data by the single-chip microcomputer.
Driverless device	Motor module Beidou positioning module Bluetooth module	The Bluetooth module controls the car movement, and the Beidou positioning module locates the car position in real time.
Data transmission device		Transmits the detection data to the terminal.

**Table 2:** Accuracy of each sensor.

Performance Index		Index Value
Ion mobility	Critical value	0.4 cm^2^/(V s)
	Maximum allowable error	±10% (ion mobility ≥0.4 cm^2^/(V s))
Air anion concentration	Measuring range	10 cm^−3^ (in the range of 10 to 5 × 10^5^ cm^−3^)
	Measurement error	±15% (in the range of 10 to 5 × 10^5^ cm^−3^)
Single measurement interval		5 min
Sampling air velocity error		≤10%
Variation of polarization voltage between plates		≤10%
Allowable error of plate clearance		≤10%
Sampling frequency		≥6 min^−1^
A/D conversion circuit		≥16
Real-time clock error		≤15 s/m
Data storage time		≥10 days
